# Proteomic Insights Into Susceptibility and Resistance to Chronic-Stress-Induced Depression or Anxiety in the Rat Striatum

**DOI:** 10.3389/fmolb.2021.730473

**Published:** 2021-10-05

**Authors:** Xiao Cai, Chen Yang, Jin Chen, Weibo Gong, Faping Yi, Wei Liao, Rongzhong Huang, Liang Xie, Jian Zhou

**Affiliations:** ^1^ Basic Medical College, Institute of Neuroscience, Chongqing Medical University, Chongqing, China; ^2^ Department of Neurology, The First Affiliated Hospital of Nanchang University, Nanchang, China; ^3^ Statistics Laboratory, ChuangXu Institute of Life Science, Chongqing, China; ^4^ Chongqing Institute of Life Science, Chongqing, China; ^5^ Department of Neurology, The Second Affiliated Hospital of Nanchang University, Nanchang, China

**Keywords:** anxiety, chronic stress, depression, proteomics, striatum

## Abstract

Chronic stress is a key factor for the onset of anxiety and depression disorders. However, the stress-induced common and unique molecular basis of the two psychiatric disorders is not fully known and still needs to be explored. Previously, we employed a chronic mild stress (CMS) procedure to induce a rat model including depression-susceptible (Dep-Sus), anxiety-susceptible (Anx-Sus), and insusceptible (Insus) cohorts. In this work, we continuously analyze the striatal proteomes of the three stressed cohorts by the use of comparative proteomics and bioinformatics approaches. Through isobaric tags for relative and absolute quantitation (iTRAQ)-based analysis, 386 abnormally expressed proteins in total were identified. These deregulated proteins are involved in various biological functions and significant pathways that are potentially connected with resistance and susceptibility to CMS-caused anxious- or depressive-like behaviors and, hence, could act as suggestive protein targets. A further parallel reaction monitoring-based independent investigation shows that alterations in Pak5, Dgkg, Scn4b, Rb1cc1, and Acin1; Ggps1, Fntb, Nudt19, Ufd1, and Ndufab1; and Dnajb12, Hbb2, Ap2s1, Ip6k1, and Stk4 were specifically connected with Dep-Sus, Anx-Sus, or Insus groups, respectively, potentially indicating that identical CMS treatment results in the different changes in the striatal protein regulations. Overall, our current proteomics study of the striatum provides an important molecular foundation and comprehensive insights into common and specific deregulations correlated with pathophysiological mechanisms that underlie resistance and susceptibility to chronic stress–induced anxiety or depression.

## Introduction

Anxiety and depression are believed to be two highly prevalent psychiatric illnesses and significant public mental health problems. Their etiologies and pathophysiologies remain incompletely investigated so far. Extensive evidence demonstrates that environmental factors, such as chronic and life event stress, are strongly associated with the risk of depression and anxiety and, thus, potentially underlie the etiologies of the two disorders (Chang and Grace, 2014; Jefferson et al., 2020). However, even with stress exposure, many people do not develop any abnormal psychiatric symptoms (Henningsen et al., 2012). To explore the potential biological mechanisms of the diseases, environmental factors, such as chronic mild stress (CMS), are commonly utilized to induce depressive and anxiety traits in animal models (Henningsen et al., 2012; Chang and Grace, 2014; Gaspar et al., 2021).

As stress-related psychiatric disorders, depression and anxiety clinically show different basic symptoms but tend to be characterized simultaneously (Liu et al., 2021; Thorp et al., 2021). Owing to the strong overlap in terms of comorbidity and pathophysiology, most of the preclinical and clinical data are frequently mixed, potentially obscuring the understanding of molecular mechanisms contributing to the development of these disorders (Chiba et al., 2012; Lucassen et al., 2016; Oh et al., 2020). Thereupon, research in the fields of depression and anxiety are increasingly focused on the separate investigation of non-comorbid subjects to find out the shared and specific nervous system features (Lotan et al., 2014; Hamilton et al., 2015; Zhao et al., 2017; Chen et al., 2018).

Existing studies show that the striatal area is most robustly implicated in subjects with symptoms of depression and anxiety (Guyer et al., 2012; Gabbay et al., 2013; Admon et al., 2015). Human neuroimaging data for depression and anxiety indicate abnormal striatal responses that may account for deregulated reward processing and corroborate the significant role of the striatum within the reward circuitry (Nikolaus et al., 2010; Guyer et al., 2012; Gabbay et al., 2013; Admon et al., 2015). Striatal dopamine D2/3 receptor-mediated neurotransmission is also reported to be involved in the pathophysiology of anxiety and depression (Schneier et al., 2008; Nikolaus et al., 2010; Leggio et al., 2015; Admon et al., 2017; Peciña et al., 2017). The striatum may be a structure in which stress and reward processing interact (Admon et al., 2015; Ullmann et al., 2020). Specifically, stress, on the other hand, reduces the activation of the striatum in response to reward and elicits robust dopamine release in the striatum on the other (Martin-Soelch et al., 2021). Hence, blunted striatal response to reward has been considered as a potential endo-phenotype associated with depression and anxiety (Luking et al., 2016). The striatum is a promising neural substrate for the interaction between stress and reward dysfunction in the two disorders (Guyer et al., 2012; Gabbay et al., 2013). Thus, a comprehensive appreciation of the striatal molecular basis, particularly the commonality and specificity of depression and anxiety is significant and useful for gaining more understanding of the pathophysiological mechanisms.

In our prior work, we constructed the CMS rat model to gain the depression-susceptible (Dep-Sus), anxiety-susceptible (Anx-Sus), and insusceptible (Insus) cohorts and meanwhile conducted a comparative proteomic analysis of the rat hippocampal tissues (Tang et al., 2019). To continuously explore the phenotype-related molecular profiles of stress-related brain regions, the rat striatal tissues from the identical batch of the CMS model were used in this work. We comparatively investigated the striatal proteomes of the Dep-Sus, Anx-Sus, Insus, and control (Ctrl) groups. The obtained proteomic data provide a molecular underpinning linking to maladaptation and adaption behavioral phenotypes, and further furnish insights into the common and unique molecular mechanisms that underlie vulnerability and resistance to stress-caused anxiety or depression.

## Materials and Methods

### CMS Rat Model and Striatal Tissue Collection

For this study, the Ethics Committee of Chongqing Medical University approved the animal protocols. Male albino Sprague–Dawley rats weighing about 250 g were obtained from the local animal center and cared for in accordance with the National Institutes of Health guidelines for the use of experimental animals. Rats were individually housed and maintained on a 12/12 h light/dark cycle with food and water provided *ad libitum*. As previously reported (Tang et al., 2019), the CMS procedure was utilized to construct the rat model. Following exposure to the 8-weeks CMS, the stressed rats were divided into the three groups: 1) Dep-Sus group [evaluated by sucrose preference (SP) and forced swimming (FS) tests], 2) Anx-Sus group [assessed by elevated plus maze (EPM) test], and 3) Insus group. Additional nonstressed rats served as the Ctrl group. A more detailed description was previously published (Tang et al., 2019). After the behavior tests, the experimental rats were decapitated, and their brains were removed. The striatal tissues were carefully separated from the brain, frozen rapidly in liquid N_2_ and kept at −80°C until further processing.

### Protein Extraction and Digestion and Peptide Labeling

Proteins were extracted from the striatal tissue samples through the use of homogenization. SDT lysis buffer containing 4.0% SDS, 0.1 M Tris–HCl, 0.1 mM dithiothreitol, and protease inhibitors (pH 8.0) was employed. The extracts were boiled for 5 min and centrifuged at 40,000 × g for 10 min. The protein contents in these supernatants were examined via Pierce bicinchoninic acid assay kit.

The extracted proteins were then digested by filter-aided sample preparation–based approach with 10-kD ultrafiltration centrifuge tubes as previously described (Gong et al., 2021). In brief, the samples were diluted by the addition of urea buffer (8 M urea, 0.15 M Tris-HCl, pH 8.0). The denatured protein samples were alkylated with 0.05 M iodoacetamide in the dark for 30 min. The samples were centrifuged and washed twice with urea buffer and then digested with trypsin at 37°C overnight in the ultrafiltration device. The tryptic digests were collected by repeated centrifugation and washed and then dried in a speed vacuum centrifuge.

Afterward, tags were used to label the tryptic peptides, strictly following the manufacturer’s instructions of the iTRAQ Reagents 8-plex kits. The total eight peptide samples from the stressed cohorts and the non-stressed Ctrl group were labeled by use of the kits 113–121. For each sample, the striatal proteins from two or three animals in each group were mixed with an equal amount (Lenselink et al., 2015). Two samples in each group were used for biological replication.

### High-pH Reversed-phase Liquid Chromatography (RPLC) Fractionation, Liquid Chromatography-Tandem Mass Spectrometry (LC-MS/MS) and MS Data Analysis

The iTRAQ-labeled peptides were mixed and then subjected to fractionation. The off-line high-pH RPLC with an 80-min linear gradient elution of 5–38% high-pH buffer containing 90% acetonitrile and 0.01 M ammonium formate (pH 10.0). The flow rate was set at 0.3 ml/min. Eluted fractions were collected as 16 fractions and vacuum concentrated.

For the following MS analysis, the peptide fractions were reconstituted in 0.1% formic acid. The peptides were analyzed by a ThermoFisher Q-Exactive Orbitrap mass spectrometer equipped with a Nanospray Flex ion source. The peptide mixture was loaded into a nanoViper C18 trap column (3 μm, 100 Å) and then separated on Thermo Scientific Easy-nLC 1200 system along with an analytical C18 column (3 μm, 75 μm × 250 mm, 100 Å). The used binary buffer system consisted of 0.1% formic acid (solution A) and 80% acetonitrile in 0.1% formic acid (solution B) under a total of 50 min linear gradient of 5–38% solution B. Data-dependent acquisition (DDA) mode was employed to acquire tandem MS data. The ion spray voltage was set to be 2 kV, and the interface heater temperature was maintained at 275°C. The m/z scan range was 350 to 2,000 for full scan, and peptides were detected in the Orbitrap at a resolution of 70,000 and a maximum injection time of 100 ms. Up to 20 precursor ions were selected for MS/MS scan using a normalized collision energy (NCE) setting of 32 and a maximum injection time of 50 ms. Only spectra with a charge state of two to five were selected for fragmentation using higher energy collisional dissociation. Dynamic exclusion for selected ions was 25 s. Automatic gain control (AGC) was set at 8E3.

As previously described (Gong et al., 2021), the identification and quantification of peptides and proteins were constituted using the ThermoFisher Proteome Discoverer (version 2.1) tool with Sequest HT search engine based on the UniProt rat database. The search parameters were set as the following: trypsin; missed cleavages, two; fixed modifications, iTRAQ 8plex on N-term and Lys, and Carbamidomethylation on Cys; variable modification, Oxidation on Met, deamidation on Asn and Gln, and Pyro-Glu, and acetylation on protein N-term; precursor mass tolerance of 10 ppm; fragment mass tolerance of 0.05 Da. The data were further screened based on the peptide-spectrum-match false discovery rate below 0.01 for protein and peptide identifications with the percolator module. Reporter ions quantifier and peptide and protein quantifier nodes was used to compute and quantify the relative ratios of peptides and proteins across samples. The ratio data was analyzed with a two-tailed Student’s *t*-test. Those proteins having a change greater than 1.2-fold and *p*-value lower than 0.05 were considered to be significantly differential as used in previous studies (Tian et al., 2018; Zhang et al., 2021). The raw data have been deposited to the ProteomeXchange Consortium (http://proteomecentral.proteomexchange.org) via the iProX partner repository with the data set identifier PXD026107 (Ma et al., 2019).

### Bioinformatics Analysis

For bioinformatics analysis, gene ontology (GO) enrichment and functional classification were constituted with the OmicsBean tool (http://www.omicsbean.cn/) (Tang et al., 2019). GO categorization (http://www.geneontology.org/) of the proteins was conducted, including biological process (GO-BP), cellular compartments (GO-CC), and molecular function (GO-MF). Pathways were identified with Kyoto Encyclopedia of Genes and Genomes (KEGG, http://www.genome.jp/kegg/) with a *p*-value of lower than 0.1 considered significant as previously reported (Son et al., 2015). The functional protein–protein interaction (PPI) network was explored in Search Tool for the Retrieval of Interacting Genes/Proteins (STRING) and visualized with Cytoscape as described previously (Gong et al., 2021).

### Parallel Reaction Monitoring (PRM) MS Assay

For PRM analysis, the striatal proteins were extracted and digested as described in the iTRAQ experiment. The resulting peptides were collected and analyzed with the Q-Exactive mass spectrometer coupled online to Easy-nLC 1200 system. The peptide mixture was separated using a 90-min linear gradient of 5–38% solution B and then analyzed using an acquisition method that combined a full-scan event with the following PRM scans as triggered by a scheduled inclusion list (Supplementary Table S1). The list contained the target precursor ions and its relative retention time based on the DDA data set with scan window ±4 min. The settings of full scans were as follows: 350–1200 m/z mass selection, 70,000 resolution (at 200 m/z), AGC target value of 3E6, and maximum injection time of 50 ms. The parameters for PRM were set as follows: 35,000 resolution, AGC target value of 2E5, maximum injection time of 100 ms, and isolation window of 1.8 m/z. Fragmentation was performed with the NCE of 28, and MS/MS scans were acquired with a starting mass of 110 m/z. The acquired raw data were processed using the Proteome Discoverer tool for protein identification. The obtained MS data were analyzed using the Skyline tool (version 19.1). The peak areas of fragment ions were summed to obtain the relative abundances of the target peptides in each sample group. The relative abundance of target proteins among sample groups were further compared based on the abundance of the corresponding peptides. Finally, the PRM data were statistically analyzed using SPSS software with Student’s *t-*test. A *p*-value lower than 0.05 was acceptable as statistically significant. The results were presented as means ± standard error (SE).

## Results

### Comparative Analysis of the Striatal Proteomes of the CMS Model Rats

**FIGURE 1:**
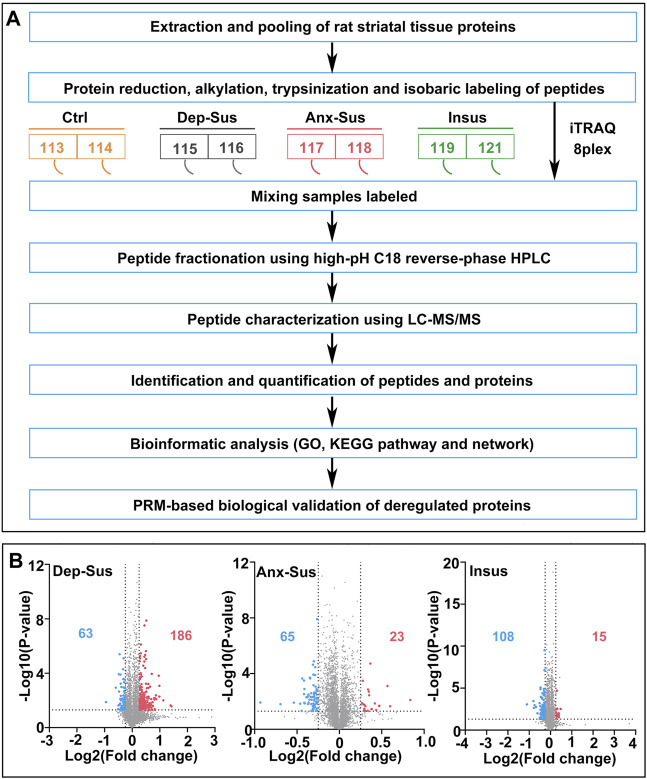
Comparative proteomic analysis of the striatum in the CMS model rats. **(A)** Flow diagram of quantitative proteomics. The striatal tissue samples from the four groups [including depression-susceptible (Dep-Sus), anxiety-susceptible (Anx-Sus), insusceptible (Insus), and control (Ctrl)] were used. **(B)** Volcano plots of abnormally expressed proteins in the three stressed groups. Blue spots represent downregulated proteins, and red spots represent upregulated proteins. Gray spots represent nondifferentially expressed proteins.

In the present work, the used rat striatum samples were from the identical batch of the CMS model in our prior study (Tang et al., 2019). Through the assessments of SP, FS, and EPM tests, we finally gained a subset of the Dep-Sus, Anx-Sus, Insus, and Ctrl groups. Next, we comparatively characterized the global expression changes in the rat striatal proteomes of the three stressed groups following the previously-used quantitative approach (Gong et al., 2021). As described in Figure 1A, an eight-plex iTRAQ experiment was designed and performed on the five animals from the Dep-Sus, Anx-Sus, Insus, and Ctrl groups. As illustrated previously (Lenselink et al., 2015), the protein extracts from two to three animals per group were equally pooled for the iTRAQ analysis. The striatal proteins from the four groups were trypsin digested and assigned to the eight isobaric labels to offer the relative quantitation. The iTRAQ-labeled peptides were mixed and characterized by LC-MS/MS. In total, 4734 nonredundant proteins were relatively quantified across all eight sample pools based upon the FDR less than 0.01. The relative protein abundances were analyzed using the signature ion ratio. Through comparing the striatal proteomes of the three stressed groups and Ctrl group, a total of 386 differentially expressed proteins were identified based upon the ratios greater than 1.2 and *p*-values lower than 0.05 as listed in Supplementary Table S2. Among them, we found that there were 186 upregulated and 63 downregulated proteins in the Dep-Sus group, 23 upregulated and 65 downregulated proteins in the Anx-Sus group, and 15 upregulated and 108 downregulated in the Insus group (Figure 1B). In the present study, we treated all proteomics data as independent hypotheses and did not adjust for multiple comparisons as illustrated in previous studies (Baughman et al., 2001; Andrews et al., 2004; Scagliotti et al., 2008; Bonati et al., 2015). The results with *p*-value < 0.05 can be taken to be suggestive of trends in striatal protein expression alterations (Zhang et al., 2021). A *p*-value smaller than this would correspond to a stronger suggestion. Herein, the proteomics profile of the striatum was compared with that of the hippocampus from our previous study (Tang et al., 2019) (Supplementary Figure S1). Although the overall proteins quantified in both the hippocampus and striatum indicate a similar profile with a 42–78% overlap, the sets of deregulated proteins of each area were considerably divergent, suggesting that the two areas had different proteomic responses to the CMS.

### Functional Characterization and Network Map of the CMS-Induced Deregulated Striatal Proteins

**FIGURE 2:**
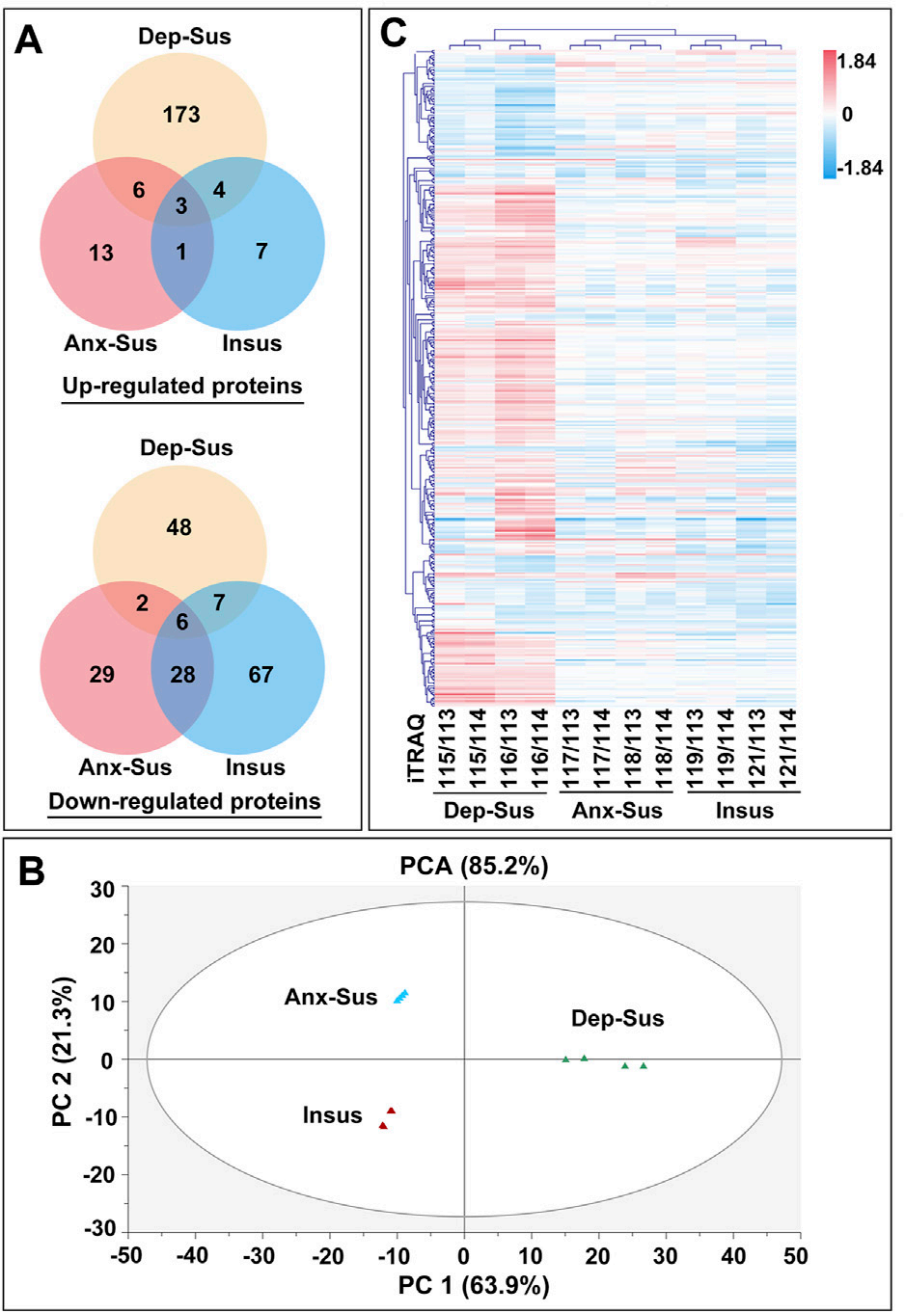
Expression analysis of the abnormally expressed proteins identified in the depression-susceptible (Dep-Sus), anxiety-susceptible (Anx-Sus), and insusceptible (Insus) groups. **(A)** Venn diagrams displaying the number of abnormally expressed proteins. **(B)** PCA of the abnormally expressed proteins based on their fold changes in the three stressed groups relative to the control (Ctrl) group. **(C)** Hierarchical clustering of the abnormally abundant proteins representing log2 of their fold changes in the three stressed groups in relative to the Ctrl group. Red represents upregulation, and blue represents downregulation with darker shades indicating greater changes in expression. The eight isobaric tags used are shown.

Our identified differential results, summarized as Venn diagrams in Figure 2A, reveal that the Dep-Sus behavioral phenotype is much more closely related to the upregulation of protein groups as a potentially maladaptive response. It was observed that there were 17 similarly deregulated proteins in the two susceptible cohorts. They might be some representative common protein deregulations between anxiety and depression. Moreover, we found that there were 49 similarly deregulated proteins among the CMS-insusceptible and the two CMS-susceptible groups, which potentially represented the results only as responsive to CMS. Interestingly, we could observe that up to 88% of these deregulated proteins were uniquely correlated with the three different phenotypes. Further, principal component analysis (PCA) and the heat map–based hierarchical cluster analysis were conducted. The PCA of all quantified proteins did not show a good separation and clustering for the three groups (Supplementary Figure S2). The PCA and heat map of the deregulated proteins suggest that these experimental samples were divided into three different groups, also directly refracting the three behavioral phenotypes (Figures 2B,C).

**FIGURE 3:**
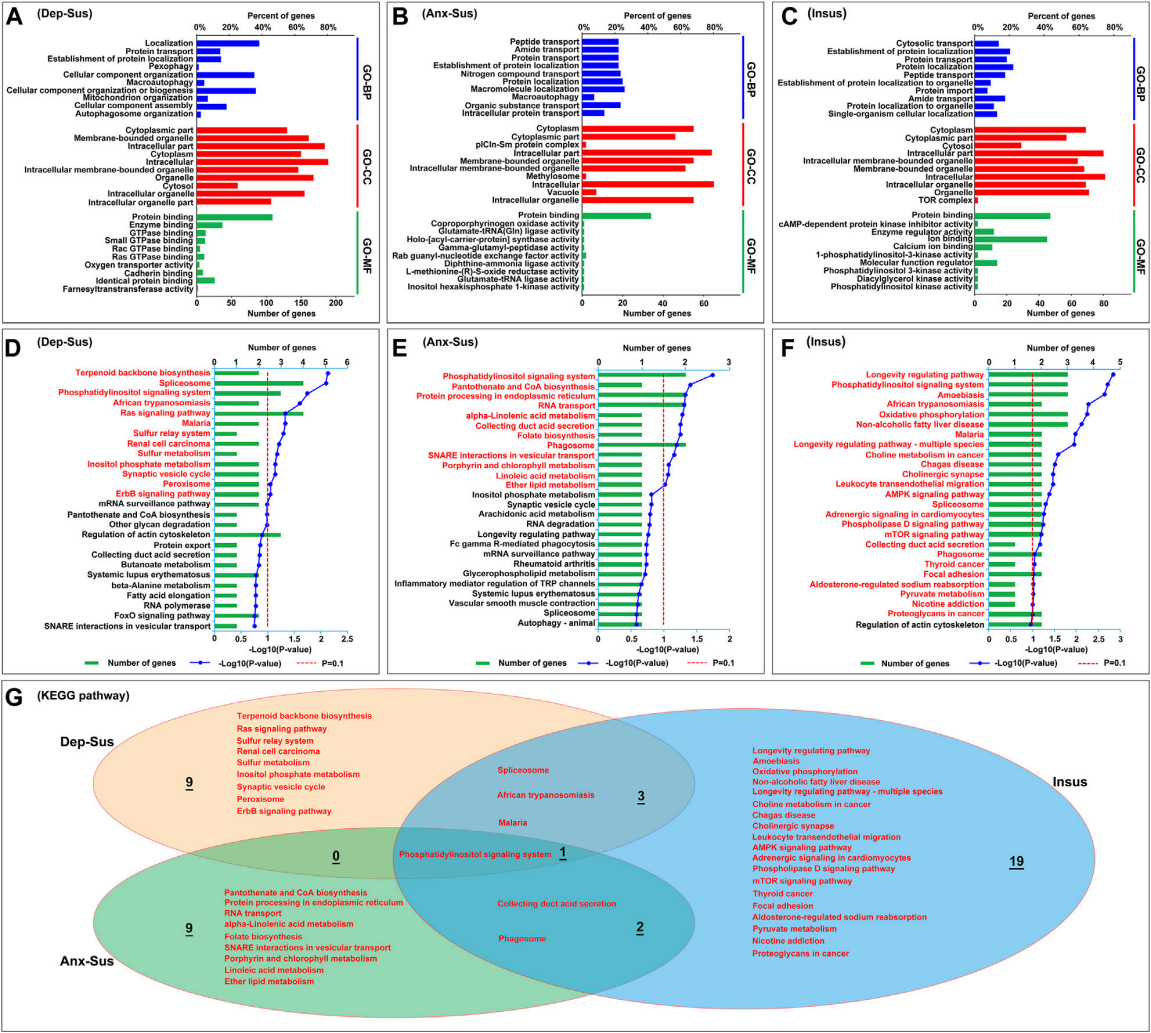
Analysis results of the GO and KEGG pathway enrichments for abnormally expressed proteins. **(A–C)** The top 10 most significant enriched entries in the three categories, including biological process (GO-BP), cellular component (GO-CC), and molecular function (GO-MF) were displayed in the depression-susceptible [Dep-Sus, **(A)**], anxiety-susceptible [Anx-Sus, **(B)**], and insusceptible [Insus, (**C**)] groups. The terms in the same category are sorted using the *p* value. **(D–F)** KEGG pathway with significant protein enrichment. The pathways that are significantly enriched in the Dep-Sus **(D)**, Anx-Sus **(E)**, and Insus **(F)** groups were shown using titles in red. **(G)** Venn diagram showing the common and unique KEGG pathway terms in the three groups.

To systematically identify the potential biological functions and processes of these abnormally expressed proteins, the OmicsBean tool was used as described previously (Tang et al., 2019). The 249 abnormally expressed proteins in the Dep-Sus group were analyzed through the use of the GO and KEGG pathway databases (Supplementary Table S3). A total of 429, 142, 159, and 13 items in the GO-BP, GO-CC, GO-MF, and KEGG pathway categories were significantly enriched. The top 10 enriched GO items are shown in Figure 3A. The GO-BP category analysis indicates that many proteins are associated with the localization, protein transport, pexophagy, macroautophagy, cellular component organization, biogenesis and assembly, and mitochondrion/autophagosome organization. The majority of GO-CC category proteins belong to the cytoplasmic and intracellular parts, organelle and cytosol. GO-MF category analysis displayed that most of the proteins were involved in protein/enzyme binding and enzyme/transporter activities. The KEGG pathway analysis indicated that the deregulated proteins were primarily involved in terpenoid backbone biosynthesis, spliceosome, signaling pathways, metabolism, and the synaptic vesicle cycle (Figure 3D).

At the same time, the GO and KEGG pathway of the 88 deregulated proteins in the Anx-Sus group were enriched. In the aggregate, 248 GO-BP, 88 GO-CC, 54 GO-MF, and 12 KEGG pathway items were significantly enriched. The 10 leading enriched GO items are indicated in Figure 3B. The GO-BP category analysis shows that the majority of proteins are correlated to transport and localization of various molecules and macroautophagy. GO-CC category analysis displayed most proteins belonging to cytoplasmic and intracellular parts, organelle, methylosome, and vacuole. GO-MF category analysis indicated that most proteins were engaged in protein binding and enzyme/factor activities. Enrichment of KEGG pathway terms showed that these deregulated proteins mainly participated in the signaling system, biosynthesis/metabolism/secretion, protein processing, transport, and phagosome (Figure 3E).

Afterward, we also performed the GO and KEGG pathway enrichment of the 123 deregulated proteins in the Insus group. In total, 248 GO-BP, 91 GO-CC, 85 GO-MF, and 25 KEGG pathway items were significantly overrepresented. The top 10 enriched GO items are shown in Figure 3C. GO-BP category analysis indicates that most of these proteins were involved in cytosolic/protein/peptide/amide transport, protein and cellular localization, and protein import, and GO-CC shows that a great part of proteins belong to the cytoplasmic and intracellular parts, organelle, cytosol, and TOR complex. Most of these proteins in the GO-MF category were engaged in protein/ion binding and inhibitor/regulator/enzyme activities, and KEGG pathway enrichment indicated that the abnormally expressed proteins were mainly involved in the longevity regulating pathway, signaling system, amoebiasis, oxidative phosphorylation, and synapse/signaling/metabolism pathways (Figure 3F).

**FIGURE 4:**
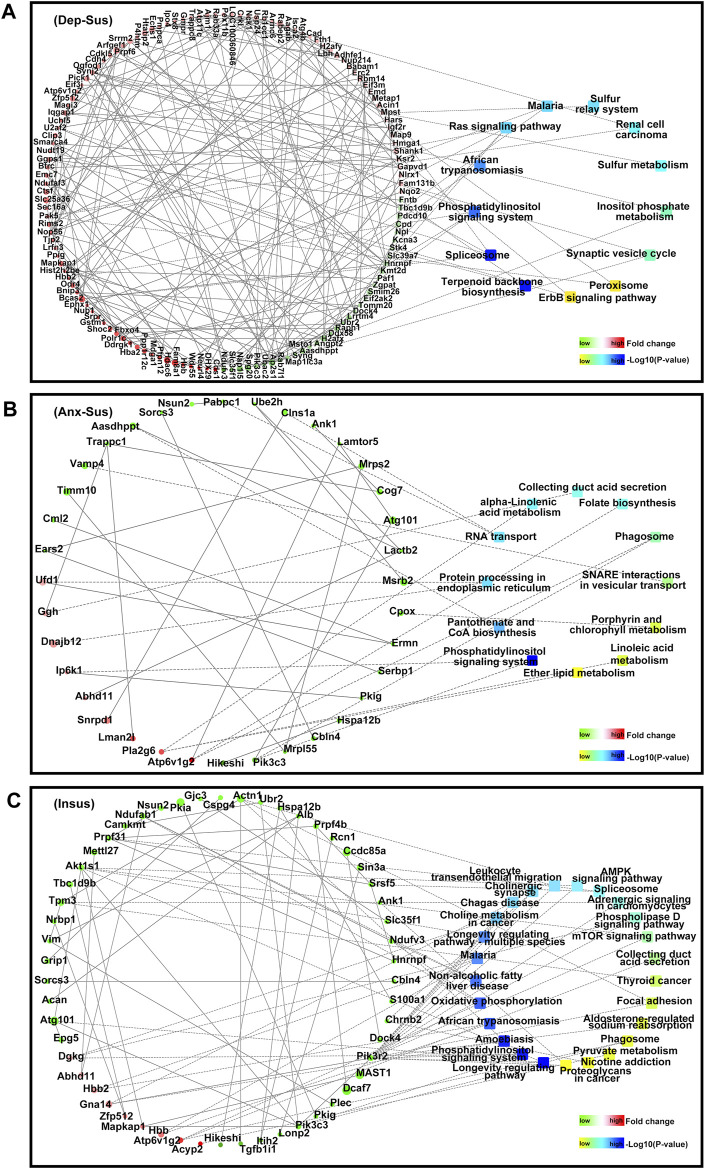
PPI network map of the abnormally expressed proteins from the three groups [Dep-Sus, **(A)**], anxiety-susceptible [Anx-Sus, **(B)**], and insusceptible [Insus, **(C)**]. The PPI networks were built based upon changes of protein expressions and enrichments of KEGG pathway terms. Circular nodes represent proteins/genes, colored in green (downregulation) and red (upregulation). Their sizes are changed based on the negative log10 of *p*-values corresponding to protein expression changes. The rectangles represent KEGG pathways, colored with gradient colors from yellow (smaller *p*-value) to blue (larger *p-*value). The interactions between proteins/genes are indicated by solid lines or are otherwise displayed by dashed lines.

Overall, of these significantly enriched KEGG pathways, six were observed to be common among the three groups (Figure 3G). Importantly, the 9, 9, and 19 pathways were found to be uniquely associated to the Dep-Sus, Anx-Sus, and Insus groups, respectively, potentially suggesting the three different biological processes as a response to CMS. Further, the proteome-inferred PPI was investigated as shown in Figures 4A–C. The networks of the three chronic-stressed cohorts were mapped through these abnormally expressed protein patterns along with the corresponding significant pathways. Based upon a unified framework of concept, we predicted 136, 36, and 54 proteins as important nodes in the networks of the Dep-Sus, Anx-Sus, and Insus groups, respectively. The network mapping uncovered a close interrelation between the deregulated proteins and the significant pathways, effectively exhibiting an interactome pool connected with the three phenotypes.

### PRM-Based Analysis of CMS-Response Proteins

**FIGURE 5:**
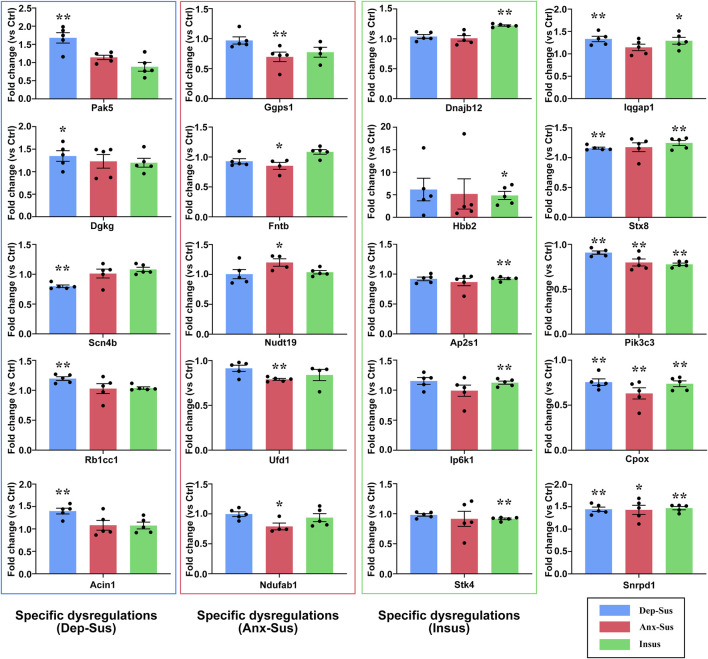
PRM analysis of the abnormally expressed proteins in the three groups (Dep-Sus, Anx-Sus, and Insus) compared with the control (Ctrl) group. Pak5, Dgkg, Scn4b, Rb1cc1, Acin1, Ggps1, Fntb, Nudt19, Ufd1, Ndufab1, Dnajb12, Hbb2, Ap2s1, Ip6k1, Stk4, Iqgap1, Stx8, Pik3c3, Cpox, and Snrpd1 were examined on the striatal protein extracts of the rats (*n* = 4–5 per group). *p < 0.05, **p < 0.01.

In this work, 20 abnormally expressed proteins of interest from the significant pathways and networks were selected to be further independently validated using the PRM MS assay. Another reason for the selection was that these proteins have not been reported or are rarely reported in previous depression and anxiety studies. In the main, the PRM data mirrored those results in the iTRAQ-based analysis (Supplementary Figure S3). There existed some discrepancies between these two quantitative results as illustrated in other proteome works (Abdi et al., 2006; Cheng et al., 2011; Xu et al., 2012; Wu et al., 2019). In addition to the determination difference of these two approaches, another cause might be the additional mixing step in the iTRAQ proteomic experiment (Xu et al., 2012; Wu et al., 2019). Compared with the Ctrl group, the expression level of Scn4b was significantly downregulated, and Pak5, Dgkg, Rb1cc1, and Acin1 were upregulated in the Dep-Sus group; the levels of Fntb, Ggps1, Ufd1, and Ndufab1 were significantly downregulated, whereas Nudt19 was upregulated in the Anx-Sus group; the levels of Ap2s1 and Stk4 were significantly downregulated, whereas Hbb2, Ip6k1, and Dnajb12 were upregulated in the Insus group (Figure 5 and Supplementary Table S1). In addition, the expressions of Iqgap1 and Stx8 were displayed to be elevated in the Dep-Sus group as compared with the Ctrl group. The levels of Pik3c3 and Cpox were reduced, and the level of Snrpd1 was elevated in the three stressed cohorts compared with the Ctrl group.

## Discussion

Chronic stress is recognized as an important risk factor in the onset of depression and anxiety disorders (Henningsen et al., 2012; Chang and Grace, 2014). Accordingly, the CMS procedure is commonly utilized to induce depressive- and anxious-like traits in experimental rodents (Henningsen et al., 2012; Chang and Grace, 2014). In our prior work, the three different phenotypes (Dep-Sus, Anx-Sus, and Insus) of rats were identified through the use of the CMS protocol based upon behavioral tests (Tang et al., 2019). This model provided a valuable and useful tool to define stress-induced molecular profiles correlated to vulnerability and resistance to anxiety or depression. Molecular investigations connected with these three phenotypes would furnish the preexisting comprehension of the underlying mechanisms of anxiety and depression with new insights, thus contributing toward translational study.

Converging evidence suggests that the striatum as one of the important neural substrates is disturbed in the reward circuitry of depression and anxiety by stress (Admon et al., 2015; Ullmann et al., 2020; Martin-Soelch et al., 2021). In the present work, the striatal site-specific proteomic signatures of the three stressed and Ctrl groups were comparatively determined through the use of an iTRAQ-based quantitation strategy. As a result, 386 abnormally expressed proteins in total were recognized in the striatum of Dep-Sus, Anx-Sus, and Insus groups compared with the Ctrl group. Intriguingly, we found that there were many abnormally expressed proteins specifically connected with the three behavioral phenotypes. The PCA and heat map of the deregulated proteins also systematically identified that the stressed groups had three different dysfunctional profiles of protein groups. This suggests that these stressed cohorts exhibit different molecular manifestations under the condition of the same CMS.

Subsequently, the integrated analysis of proteomics findings with bioinformatics tools identified many significant pathways in the striatum uniquely associated with the three varied phenotypes. The happened protein deregulations and activated biological pathways in the stressed cohorts likely had great differences, potentially suggesting that the rats displayed different neurobiological responses to CMS. The subsequent network map unraveled abnormal protein systems and multiple significant pathways connected with resistance and vulnerability to stress-caused anxiety or depression.

In this work, we further conducted the PRM MS assay to independently validate the 20 abnormally expressed proteins involved with the significant functions and pathways. The obtained results indicate that Pak5, Dgkg, Scn4b, Rb1cc1, and Acin1 were uniquely deregulated in the striatum of the Dep-Sus group, and Ggps1, Fntb, Nudt19, Ufd1, and Ndufab1 were uniquely deregulated in the Anx-Sus group. These expression responses distinctly in the striatum indicate that the same stimulus would lead to the different protein dysfunctions, thereby practically pushing toward varied neurobiological pathways connected with depressive or anxious phenotypes. We found that Pak5, Dgkg, Scn4b, and Rb1cc1 were involved in a variety of important signaling pathways, including ErbB, phospholipase D, adrenergic, and autophagy signaling pathways. Previously, these important signaling pathways are preliminarily implicated in depression (Daskalopoulos et al., 2012; Feng and Huang, 2013; Dang et al., 2016; Xiao et al., 2018; Kim and Leem, 2019). In the Dep-Sus group, specific alterations of these proteins would result in the disturbance of the corresponding pathways and the onset of depression-like behavior. Meanwhile, we also found that Fntb and Ggps1 were involved in terpenoid backbone biosynthesis, Nudt19 was involved in peroxisomal lipid metabolism, and Ndufab1 was involved in fatty acid biosynthesis. The disturbance of these important metabolic pathways are gradually discovered in stress-related disorders (Oliveira et al., 2015). A previous metabolic profile indicates that there are many changes in lipid metabolism in the prefrontal cortex of rats subjected to social defeat stress (Liu et al., 2017). In the Anx-Sus group, specific deregulations of these proteins would lead to abnormalities in the respective metabolic pathways. Generally, these metabolisms are considered to be an important source of energy supply. The abnormal regulation of multiple metabolic processes would potentially cause a negative energy balance in the striatum of the Anx-Sus rats (Harris, 2015). Furthermore, the expressions of Dnajb12, Hbb2, Ap2s1, Ip6k1, and Stk4 were observed to be distinctly changed in the Insus group. These proteins are suggested to be involved in essential regulatory mechanisms for the synaptic vesicle cycle, phosphatidylinositol signaling system, and Ras signaling pathway. As a part of the adaptor protein complex 2, the dysregulation of Ap2s1 in the Insus group potentially plays a significant role in the recycling process of the synaptic vesicles to sustain the normal synaptic transmission (Collins et al., 2002). To a large extent, the specific alterations of these proteins in the Insus group likely reflect a positive molecular path for coping with the CMS-caused protein regulatory abnormality for stress protection in the rat striatum (Krishnan et al., 2007; Henningsen et al., 2012). Taken together, our present comparative proteomics analysis found a series of protein deregulations in the striatum of the stressed rats. The data provided clues to the molecular mechanisms underlying the three CMS-induced behavioral phenotypes and defined multiple potential therapeutic protein targets.

However, the present study had several limitations. First, the direct correlations of the observed striatal proteomic differences with the three behavioral phenotypes were uncertain and need to be continuously investigated by using modern molecular and genetic approaches based on local brain-tissue injection combined with sophisticated behavioral assessments in the future study. Second, it is important to further explore the precise physiological or pathological roles of these candidate proteins behind such expression perturbations in the striatum.

## Conclusion

In conclusion, the present study conducted iTRAQ- and PRM-based quantitation experiments to investigate the effects of CMS on the rat striatal protein expressions. For all we know, it has not previously been reported that such striatal protein candidates were potentially related to adaptation and maladaptation to CMS-induced depression or anxiety. Our current proteomic data provides a scientific foundation for future study to explore common and specific deregulations correlated with the stress-induced different behavioral phenotypes and might lead to a better comprehension of the underlying pathophysiological mechanisms of depression and anxiety disorders.

## Data Availability

The proteomics raw data can be found in the ProteomeXchange Consortium with the identifier PXD026107. The datasets supporting this article have been uploaded as part of the electronic Supplementary Material.
