# Supplementary material for: Proteomic Insights Into Susceptibility and Resistance to Chronic-Stress-Induced Depression or Anxiety in the Rat Striatum
Source: Front Mol Biosci. 2021 Oct 5;8:730473. doi: 10.3389/fmolb.2021.730473 (PMC8523913; doi:10.3389/fmolb.2021.730473)
Supplement: Supplementary file 4 [file DataSheet1.docx]

**Supplementary information**

**Proteomic insights into susceptibility and resistance to chronic-stress-induced depression or anxiety in the rat striatum**

Xiao Cai^1^, Chen Yang^1^, Jin Chen^1,2^, Weibo Gong^1^, Faping Yi^1^, Wei Liao^1^, Rongzhong Huang^3,4^, Liang Xie^1,5,^*, Jian Zhou^1,^*

^1^Institute of Neuroscience, Basic Medical College, Chongqing Medical University, Chongqing 400016, China

^2^Department of Neurology, The First Afﬁliated Hospital of Nanchang University, Nanchang 330006, China

^3^Statistics Laboratory, ChuangXu Institute of Life Science, Chongqing 400016, China

^4^Chongqing Institute of Life Science, Chongqing 400016, China

^5^Department of Neurology, The Second Afﬁliated Hospital of Nanchang University, Nanchang 330006, China

These authors contributed equally: Xiao Cai, Chen Yang, Jin Chen and Weibo Gong

*To whom correspondence should be addressed:

Jian Zhou

Institute of Neuroscience, Basic Medical College, Chongqing Medical University, 1 Yixueyuan Road, Yuzhong District, Chongqing 400016, China. Tel: +86-23-68485763. E-mail address: zhoujian@cqmu.edu.cn (J. Zhou).

Liang Xie

Department of Neurology, The Second Affiliated Hospital of Nanchang University, 1 Minde Road, Nanchang 330006, Jiangxi, China. E-mail address: xl580122@163.com (L. Xie).

**Supplementary material**

**Supplementary Figure S1.** Comparisons of the proteomic profiles of the hippocampus and striatum. (A) Venn diagram showing the number of the total proteins quantified in each brain area. (B, C and D) The diagrams displaying the number of abnormally-expressed proteins in the depression-susceptible (Dep-Sus, B), anxiety-susceptible (Anx-Sus, C) and insusceptible (Insus, D). Hip: hippocampus; Str: striatum.

**Supplementary Figure S2.** Principal component analysis (PCA) of all quantified proteins based on their fold changes in the depression-susceptible (Dep-Sus), anxiety-susceptible (Anx-Sus) and insusceptible (Insus) groups in relative to the control (Ctrl) group.

**Supplementary Figure S3.** Comparison between isobaric tags for relative and absolute quantitation (iTRAQ)-based and parallel reaction monitoring (PRM)-based data in the depression-susceptible (Dep-Sus), anxiety-susceptible (Anx-Sus) and insusceptible (Insus) groups in relative to the control (Ctrl) group.

**Supplementary Table S1.** Parallel reaction monitoring (PRM) inclusion list and information on the target peptides and corresponding proteins. The PRM data were normalized by the iRT peptides that were added to all of the samples. The method for treating the PRM data was described in the Methods section.

**Supplementary Table S2.** Complete protein identification results from the depression-susceptible (Dep-Sus), anxiety-susceptible (Anx-Sus), and insusceptible (Insus) groups in the isobaric tag for relative and absolute quantitation (iTRAQ)-based experiment. Blue highlighted the abnormally-expressed proteins.

**Supplementary Table S3.** Complete results of gene ontology (GO) biological process (GO-BP), cellular component (GO-CC), molecular function (GO-MF), and Kyoto Encyclopedia of Genes and Genomes (KEGG) pathway enrichments for the abnormally-expressed proteins in the depression-susceptible (Dep-Sus), anxiety-susceptible (Anx-Sus), and insusceptible (Insus) groups. Blue highlighted the significantly-enriched terms.


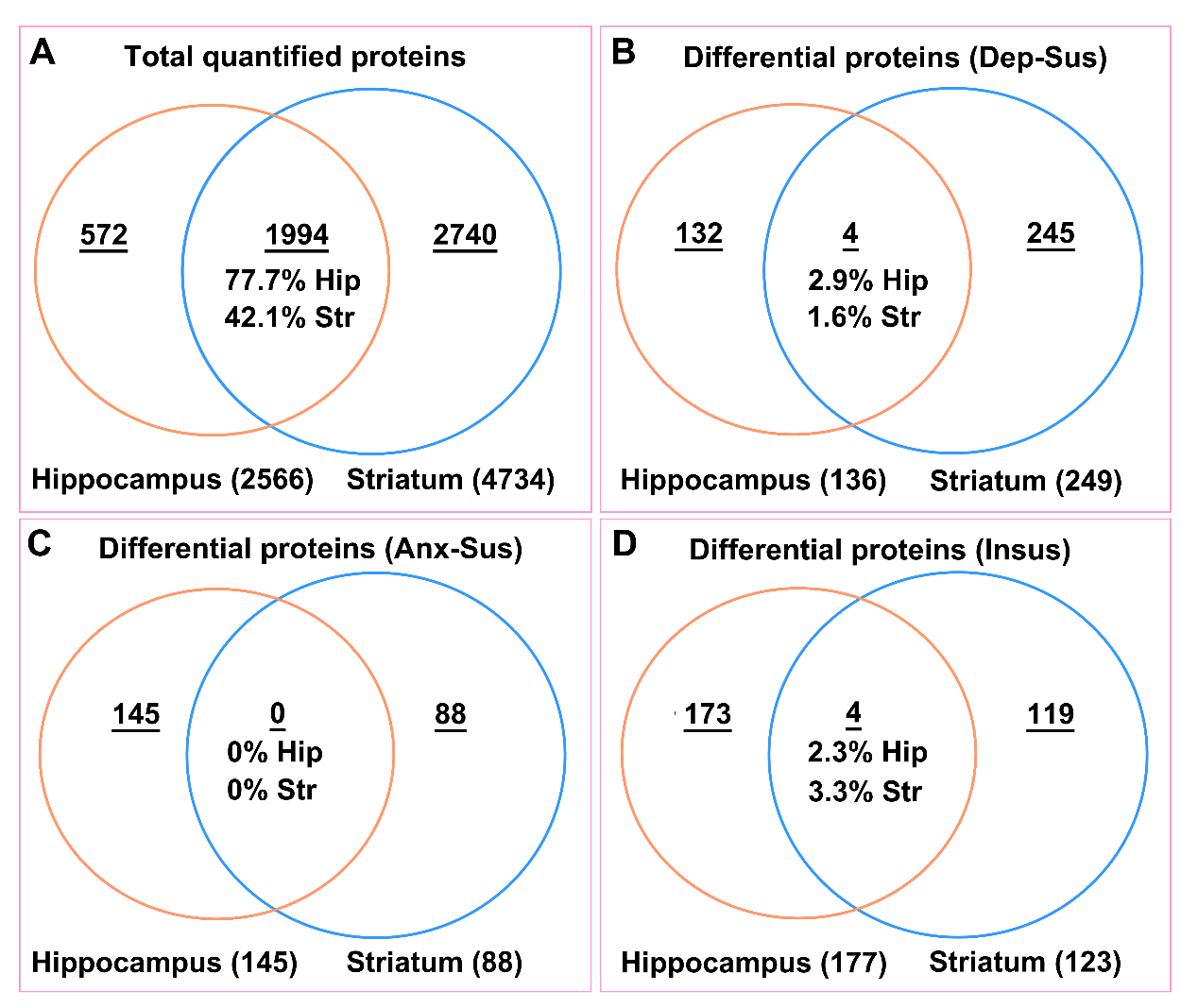


**Supplementary Figure S1**


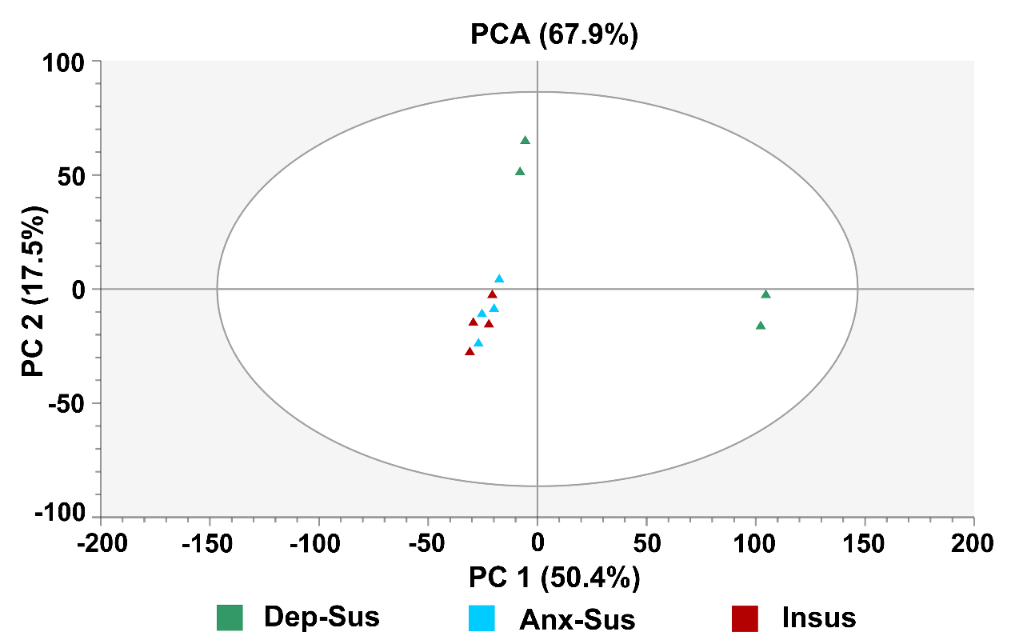


**Supplementary Figure S2**


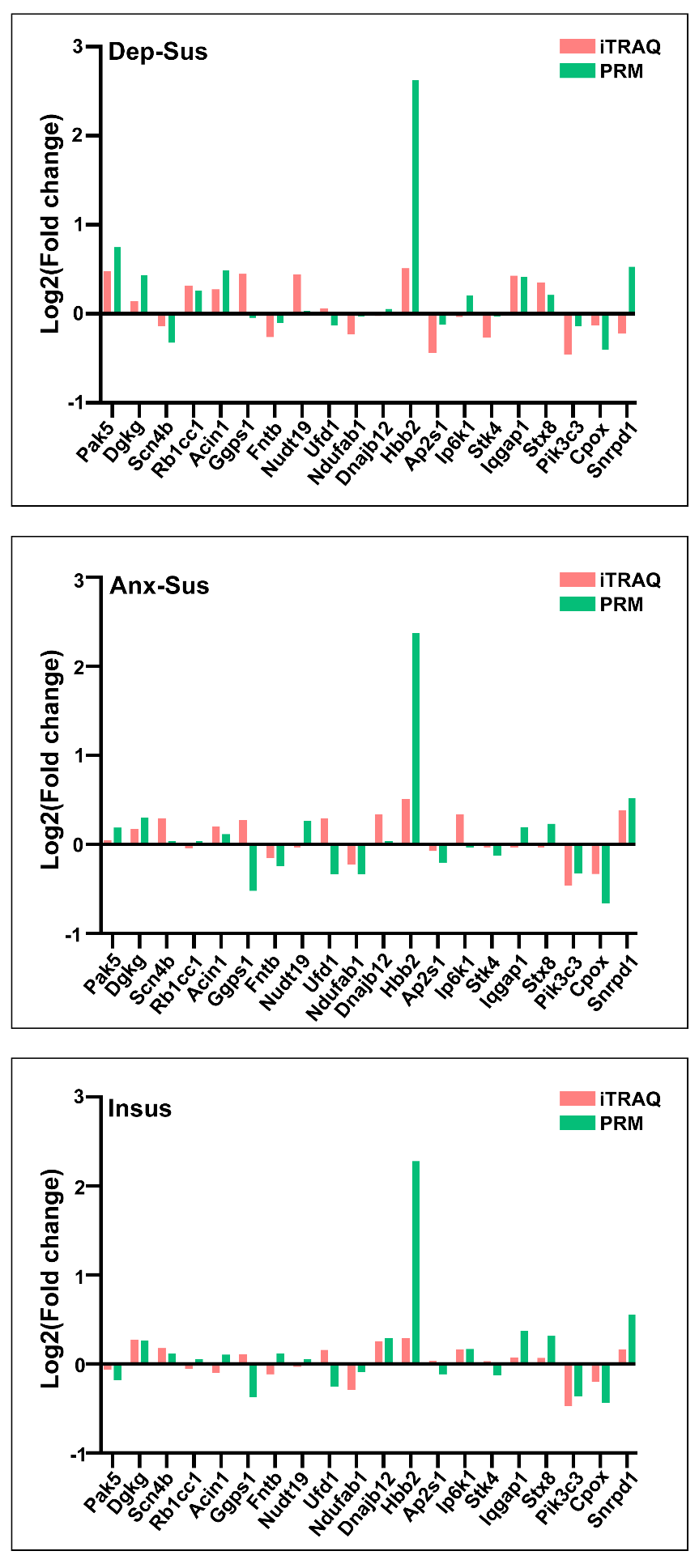


**Supplementary Figure S3**
